# CareMobi to Improve Communication Between Caregivers and Adult Day Centers for People With Dementia: Mixed Methods Feasibility Study

**DOI:** 10.2196/85090

**Published:** 2026-04-24

**Authors:** Tina Sadarangani, Laura Peralta, Shahrzad Siamdoust, Jackie Finik

**Affiliations:** 1 NYU Rory Meyers College of Nursing New York, NY United States

**Keywords:** adult day services, dementia, mobile health, community-based, feasibility, adult day care

## Abstract

**Background:**

Adult day centers (ADC) are well-positioned to address social isolation among the rapidly growing population of people living with dementia but are underused relative to other forms of long-term care. Mistrust of these centers among family caregivers remains a barrier to their use. Digital health tools offer a promising approach to enhance transparency, improve communication, and build trust between caregivers and ADCs. As such, researchers at New York University (NYU) developed CareMobi, a user-centered mobile app that supports care coordination between ADC, care providers, and caregivers.

**Objective:**

This study aimed to evaluate the real-world usability, acceptability, and feasibility of CareMobi among staff in ADCs and the caregivers of people living with dementia who attend centers.

**Methods:**

Guided by the Goal–Question–Metric (GQM) framework, we conducted a low-burden field usability evaluation of CareMobi in 2 ADCs. Data included baseline caregiver surveys, app usage logs, administration of the **Post-Study System Usability Questionnaire**, and participant interviews. Feasibility benchmarks were established a priori using STOP-AMEND-GO criteria for evaluable retention, usability, satisfaction, engagement, and acceptability. Quantitative analyses summarized demographics, caregiver confidence, and app usability (7-point scale and median scores). Qualitative analyses used thematic coding of interviews and written feedback to identify perceived benefits, barriers, and recommendations for implementation.

**Results:**

Family caregivers (n=15) from 2 ADCs participated in a mixed-methods pilot. Participants were primarily female (87%), non-Hispanic White (93%), and middle-aged or older (67% aged 30-64 years). Most were sole caregivers (80%). Baseline confidence in managing dementia-related care was high, particularly in communication with providers. Of 15 enrolled caregivers, 14 (93%) logged into CareMobi at least once, and the majority “agreed/strongly agreed” that the app was easy to use (6/8, 75%) and easy to learn (7/8, 87.5%); 75% (6/8) of the participants liked the interface and found the information well organized. All staff (n=3) indicated the CareMobi app was easy to use, easy to learn, and well organized (3/3, 100%). Willingness to use the app beyond the study period was high among caregivers (6/8, 75%) and staff (3/3, 100%). Open-ended caregiver responses emphasized reassurance from daily updates, user-friendliness, and time savings. Staff reported reduced “phone tag” streamlining coordination, particularly for new ADC enrollees. However, established caregivers tended to use the app passively, defaulting to phone calls. Themes highlighted the need for structured logging guidance, role-specific templates, and integration with existing systems. Caregivers and staff noted that routine updates fostered trust, improved decision-making about attendance, strengthened communication with health care providers, and provided peace of mind.

**Conclusions:**

CareMobi offers a promising approach to modernizing ADC in ways that respect resource limitations and caregiver needs, through combining digital transparency with low-burden communication. Future efforts should center on equity and focus on linguistic inclusion, infrastructure support, and engagement in underserved communities.

## Introduction

Social isolation and loneliness are widely recognized, modifiable social determinants of health in later life. In the United States, approximately one quarter of community-dwelling adults aged ≥65 years are socially isolated and large proportions report loneliness [1]. Social isolation and loneliness are associated with a 32% and 14% increased risk of all-cause mortality, respectively. They are also linked to increased morbidity, including a 29% higher risk of coronary heart disease and a 32% higher risk of stroke [1,2]. These risks are amplified for people living with dementia and their family care partners, for whom shrinking networks, mobility limitations, and caregiver burden compound barriers to engagement in community life [3].

Adult day centers (ADCs) are a cornerstone of community-based long-term care and a promising, equity-oriented platform to counter social disconnection at scale [4], especially for people living with dementia. They represent the most diverse sector of long-term care, serving more than 250,000 predominantly low-income (72%) and ethnically diverse (58%) individuals, who are able to remain in their own homes while receiving daytime care that fosters engagement, health monitoring, and meaningful socialization. By providing structured programming, culturally responsive services, and respite for care partners, ADCs buffer social isolation and loneliness for people living with dementia, and simultaneously reduce caregiver burden [4,5]. Yet, despite the fact that more than 80% of older adults report wanting to remain in their homes [5], and that social engagement is consistently linked to better health outcomes [6], ADCs remain vastly underused compared to other, more costly forms of long-term care that either don’t offer socialization or allow people to remain at home. These include home health (serving more than 3 million) or nursing homes (with 1.2 million residents) [7].

There are several barriers that limit uptake of ADCs, including transportation challenges, cost, and limited awareness of services; but one of the most significant is trust. A key relational barrier to ADC use, particularly among socially isolated or underserved families, is mistrust of these facilities and difficulty communicating with center staff about day-to-day care [7]. Family caregivers often report uncertainty about whether staff truly understand their loved one’s needs, whether cultural preferences will be respected, and whether information will be shared transparently, all of which cause apprehension and can deter sustained engagement.

Research has consistently highlighted improved information-sharing as crucial to overcoming barriers in the uptake of respite care [8]. While many older adults can articulate their own needs, for those who are unable to do so, like people living with dementia, effective communication often depends on carers and respite staff [8]. Digital health tools, when designed for usability and embedded into real-world workflows, offer a promising strategy to enhance transparency, strengthen bidirectional communication, and improve coordination between families and community providers [9,10]. By providing a lens into the day-to-day care of their loved ones, such tools can reduce caregiver apprehension, build trust in ADCs, and allow people living with dementia to be more socially engaged by attending these centers.

A growing number of mobile health (mHealth) interventions have been developed to support dementia caregiving. such as symptom-monitoring tools, secure messaging platforms, and caregiver education apps, but few are designed for or evaluated within ADC environments, which have distinct documentation practices, staffing structures, and relational communication needs. Existing tools often focus on clinical care coordination or remote monitoring rather than the day-to-day visibility that shapes caregiver trust in ADCs and influences continued attendance. CareMobi is a mHealth app designed to strengthen communication, coordination, and transparency between ADC staff, family caregivers, and health providers. The app allows for real-time updates on daily activities, health status, and logistical information, helping caregivers interpret changes in behavior at home and enhancing continuity of care across settings. CareMobi differs conceptually from prior interventions by centering a digital trust-building approach: it uses real-time, low-burden updates to reduce caregivers’ uncertainty, strengthen relational continuity, and support socio-technical alignment between caregivers and ADC workflows. This focus on transparency, role-specific communication, and workflow feasibility represents a novel contribution to the mHealth landscape and addresses known barriers to ADC use [10-12].

We posit that by making ADC-based care more visible to families, through timely updates and low-burden, role-specific interactions, CareMobi can reduce uncertainty, foster trust, and simplify decisions about ADC attendance and sustained participation. Initial interactive prototype testing allowed us to make user-informed modifications that laid the foundation for real-world evaluation [10]. The purpose of this feasibility study was to: (1) evaluate acceptability (perceived usefulness, satisfaction, and burden for caregivers and staff), (2) assess demand (enrollment, uptake, and engagement with core features), and (3) examine implementation and practicality within ADC workflows (resources, role fit, and compatibility with existing communication practices). Our evaluation also sought to understand caregivers’ baseline confidence levels and areas where ADCs could provide additional support, as well as the platform’s potential impacts on caregivers’ overall experience and confidence in the care provided by ADCs.

## Methods

### Overview and Rationale

CareMobi was developed using a user-centered design process that actively engaged ADS staff, family caregivers, and community stakeholders throughout its development. Iterative feedback was solicited through interviews, focus groups, and usability testing to ensure the tool was feasible to integrate into workflows, responsive to caregiver needs, and adaptable across diverse ADCs. This participatory process emphasized co-design to identify priority features (eg, streamlined messaging, daily activity summaries, and health-related observations) and reduce barriers such as duplicate documentation or technology burden. The full details of CareMobi’s design and development process, including prototype testing, have been reported elsewhere in prior research; here we focus on evaluation of its implementation, usability, and acceptability in practice [10].

### Study Framework

We adopted a pragmatic, low-burden field usability evaluation of CareMobi in ADCs, guided by a Goal–Question–Metric (GQM/GAQM) [13] approach. Originally developed for software engineering and adapted for usability research, GQM ensures that data collection remains purposeful, relevant, and aligned with stakeholder needs. In practice, evaluators first articulate the overarching goals (eg, feasibility, usability, and acceptability), then derive key questions that operationalize those goals (eg, “Can caregivers complete core tasks without difficulty?”), and finally specify the metrics that can provide evidence (eg, task completion rates, satisfaction ratings, and interview themes). This hierarchical structure promotes rigor while keeping evaluations pragmatic and low burden, particularly in resource-constrained real-world settings such as ADCs. This allowed us to explicitly tie high-level goals (usability, acceptability, and feasibility) to specific questions and measurable metrics, while respecting constraints of under-resourced ADC settings. We aligned these questions and metrics with the GAQM framework’s principle of first defining a goal (conceptual), then attributes (usability dimensions), then operational questions, and finally metrics (quantitative and qualitative). This approach is outlined in Table 1.

**Table 1:** Goal–questions–metrics framework.

Goal (Usability and implementation focus)	Usability or implementation attribute	Key questions	Metrics and indicators
Assess satisfaction and perceived usability	Perceived usability and satisfaction	How do users rate the app on ease, clarity, and burden?	Likert-scale items (eg, PSSUQ^a^)
Understand barriers, user experience, and trust	Qualitative and contextual implementation	What aspects of the app or workflow did users find confusing, burdensome, or trust-enhancing? Where do they feel uncertain?	Thematic codes from semistructured interviews; free-text questionnaire comments
Engagement and uptake in real settings	Implementation and adoption	How many caregivers and staff enroll, how often do they use core features?	Enrollment counts, caregivers who use it at least once

^a^PSSUQ: Post-Study System Usability Questionnaire.

### Overall Study Design

The evaluation combined field (in situ) testing, self-user usability (unmoderated use in real settings), post-use questionnaire, and qualitative interviews with staff and family caregivers in ADCs located in rural and underserved communities.

### Setting

We partnered with 2 ADC sites primarily serving people living with dementia that were recruited at the Annual Meeting of the National Adult Day Services Association. Within each center, paid staff and family caregivers were recruited to pilot test CareMobi in their real daily workflows (as opposed to a lab setting), over a 12-week period. Our approach was consistent with “field studies” in mobile usability literature, where tasks are executed in the natural environment, and contextual factors (interruptions, real devices, ambient conditions) are preserved [10].

### Recruitment and Eligibility

Recruitment occurred over 3 months through center leadership, who distributed study information to eligible caregivers and staff; interested individuals were then contacted by the research team to confirm eligibility and obtain informed consent. Eligible ADC staff included those directly involved in the day-to-day care of people living with dementia and responsible for communicating with family caregivers. Eligible family caregivers were those who identified as the primary caregiver of people living with dementia attending one of the participating ADCs, were aged 18 years or older, and owned a smartphone or tablet capable of running the CareMobi app. Both staff and caregivers were required to speak English and be willing to provide informed consent.

### Training and Study Procedures

Prior to deployment, participants received minimal training, an intentional design choice to evaluate how intuitive the CareMobi platform was in real-world use. ADC staff participated in a 30-minute orientation covering the study overview and app functions. Family caregivers were assisted, as needed, in downloading the app using a QR code and were then encouraged to explore it independently. For ongoing support with the app, staff and caregivers were directed to a video library developed by an instructional technologist. Staff and caregivers were also advised to reach out to the study team with any questions or regarding technical issues. This pragmatic, low-burden approach mirrored real-world implementation conditions and aligned with the GQM framework, specifically addressing goals of assessing intuitiveness and efficiency of use with minimal external support.

Staff were instructed to use their discretion and share information through CareMobi that was tailored to each person living with dementia and caregivers’ needs, while caregivers were encouraged to use the app as much or as little as they preferred to engage with the ADC. There was no minimum requirement for use in order to reflect real-world engagement patterns. CareMobi uses HIPAA (Health Insurance Portability and Accountability Act)–compliant security protocols, including encrypted data transmission and storage, authenticated user access, and restricted role-based permissions. No identifiable information was shared outside the secure platform, and all study data were deidentified prior to analysis.” Following consent, participants were instructed to download the CareMobi app from their preferred app store, and family caregivers completed a baseline survey. After the 12-week usage period, participants completed a post-use questionnaire assessing perceived usability, satisfaction, burden, and open-ended feedback. We also conducted semistructured interviews with a purposive subset of caregivers and staff, focusing on their experience, trust concerns, communication challenges, and suggestions for improvement. Staggered incentives, in the form of gift cards, were provided upon completion of the baseline survey, usability questionnaire, and interview (US $150 total).

### Measures and Instruments

#### Demographics

We collected self-reported baseline demographic characteristics from family caregivers, including age, gender, role, and caregiving relationship, to contextualize study findings.

#### Caregiver Confidence

Two validated caregiver-reported instruments were used to assess caregiver experience and confidence. The Caregiver Confidence in Sign/Symptom Management (CCSM) Scale is a 4-item instrument that evaluates caregivers’ perceived ability to recognize, monitor, and respond to changes in their care recipient’s symptoms [14]. Items are rated on a 5-point Likert scale ranging from not at all confident (1) to extremely confident (5), with higher scores indicating greater confidence in symptom management. Prior research has demonstrated good internal consistency (Cronbach α=0.85) and construct validity, supporting its use in community-based caregiver populations [14].

#### Usability

To capture perceptions of usability and satisfaction, we administered the Post-Study System Usability Questionnaire (PSSUQ) [15,16], a validated 16-item scale that measures user perceptions across three domains: system usefulness, information quality, and interface quality. Items are rated on a 7-point Likert scale (strongly disagree [1] to strongly agree [7]), with lower scores indicating greater usability. The PSSUQ has been widely applied in digital health research and demonstrates high internal consistency (Cronbach α=0.83-0.96) [15,16].

### Satisfaction

Finally, a **semistructured interview guide** was used to explore participants’ experiences in greater depth. Interview domains included perceptions of transparency and trust, communication gaps, perceived benefits or frustrations, barriers to engagement, and suggestions for improving design or workflow alignment. Open-ended questions (eg, “Were there any parts of the app that felt confusing, frustrating, or difficult to use?”; “How, if at all, did it impact your experience with the ADC?”) elicited participants’ reactions to CareMobi, perceptions of usability, potential barriers, and impact on their relationship with the ADC as it pertained to the health of the people living with dementia.

### Data Analysis

#### Quantitative

The pilot was conducted as a feasibility and usability study rather than an efficacy trial, and analyses were therefore descriptive and hypothesis-generating. Quantitative analyses included calculation of descriptive statistics (frequencies, medians, and ranges) for demographic and questionnaire results.

#### Qualitative

Participant interviews lasted approximately 30 minutes and were conducted by either the principal investigator (TS), who has formal training in mixed-methods research, or a research assistant (LP) with a master’s in clinical research. Interviewers used member checking informally during data collection by pausing to confirm interpretations. All interviews were recorded, transcribed, and reviewed for accuracy. Field notes and reflexive journals were maintained to document interviewer observations, decision-making, and potential sources of bias.

Coding focused on domains relevant to the study aims, including trust and transparency, communication barriers, workflow fit, and caregiver confidence. Themes were then triangulated with quantitative findings (eg, task completion and usability scores) to provide a more comprehensive understanding of user experience. Two coders (one PhD, one undergraduate nursing student) independently coded transcripts in Dedoose, with the principal investigator reviewing a 20% subset to ensure reliability. The research team met regularly to discuss coding discrepancies, update the codebook, and validate emerging findings. Saturation was considered reached when no new categories were identified.

### Feasibility Benchmarks

To evaluate feasibility, we applied a priori STOP-AMEND-GO [17] criteria across five domains: retention, usability, satisfaction, engagement, and acceptability. **Evaluable retention** was defined as completion of the 3-month follow-up survey, excluding non–study-related attrition (death or transition to a higher level of care); thresholds were STOP if <60%, AMEND if 60-69%, and GO if ≥70%. **Usability** was assessed using the PSSQ, with criteria based on the proportion of participants endorsing “agree” or “strongly agree” for items on ease of use and ease of learning; thresholds were STOP if <60% on any item, AMEND if 60-74%, and GO if >75% on each. **Satisfaction** was measured as the percentage of caregivers and staff endorsing “agree/strongly agree” on overall satisfaction with CareMobi, with thresholds STOP if <60%, AMEND if 60-74%, and GO if >75%. **Engagement** was defined as the proportion of participants who created an account and used the app at least once; thresholds were STOP if <50%, AMEND if 50-69%, and GO if ≥70%. Finally, **acceptability** was measured as the percentage of staff and caregivers expressing willingness to continue using CareMobi beyond the study period, with thresholds STOP if <50%, AMEND if 60-74%, and GO if >75% (Table 2).

**Table 2:** Study feasibility benchmarks (STOP-AMEND-GO criteria) and results.

Domains	Metric	STOP	AMEND	GO	Study result		
					Caregiver	Staff	Criterion
Evaluable retention	3-month completion of survey excluding nonstudy-related attrition (ie, death)	<60%	60%-69%	≥70%	100%	100%	GO
Usability	% “agree/strongly agree” on easy to use, easy to learn on PSSQ	<60% on any	60%-74% on any	75% on each	75%	100%	GO
Satisfaction	% “agree/strongly agree” on overall satisfaction	<60%	60%-74%	75% on each	>75%	100%	GO
Engagement	% of caregivers who created an account and used the app at least once	<50% any-use	50%-69% any-use	≥70% any-use	93%	100%	GO
Acceptability	% of staff and caregivers expressing that they will continue to use CareMobi beyond study period	<50%	60%-74%	75%	75%	100%	GO

### Ethical Considerations

All study procedures were reviewed and approved by the Institutional Review Board at New York University (IRB-FY2023-7509). All participants provided informed consent prior to participation, and all data were deidentified to protect confidentiality. Compensation was US $150 for participation in all phases of the study.

## Results

### Caregiver Characteristics

In total, 15 caregivers completed baseline surveys (Table 3). Most participants identified as female (13/15, 86.7%), were aged 30-64 years (10/15, 66.7%), and reported some college or higher education (14/15, 93.3%). The sample was predominantly non-Hispanic White (14/15, 93.3% White; 14/15, 93.3% non-Hispanic). Most were married (11/15, 73.3%). Overall, 40% (6/15) were employed full-time and 20% (3/15) part-time; 26.7% (4/15) were retired. All respondents were family caregivers (15/15, 100%); 80% (12/15) identified as the sole caregiver. Relationship to the people living with dementia was most commonly adult child (7/15, 46.7%) or spouse or partner (5/15, 33.3%). Most had been caregiving for ≥1 year (11/15, 73.3%).

**Table 3:** Demographic characteristics of the family caregivers and staff at the adult day centers.

Characteristics			Values, n (%)	
**Family caregivers (n=15)**				
	**Sex**			
		Male		2 (13.3)
		Female		13 (86.7)
	**Age (years)**			
		30-49		4 (26.7)
		50-64		6 (40)
		65+		4 (26.7)
		Unknown		1 (6.7)
	**Education**			
		High school graduate or less		1 (6.7)
		Some college		6 (40)
		College graduate or higher		8 (53.3)
	**Race (ethnicity)**			
		Non-Hispanic White		14 (93.3)
		Other Non-Hispanic		1 (6.7)
	**Marital status**			
		Single		2 (13.3)
		Married		11 (73.3)
		Widowed		1 (6.7)
		Divorced		1 (6.7)
	**Employment status**			
		Employed full-time		6 (40)
		Employed part-time		3 (20)
		Retired		4 (26.7)
		Other		2 (13.3)
	**Annual household income (US $)**			
		<$25,000		2 (13.3)
		$25,000–$49,999		4 (26.7)
		$50,000–$99,999		6 (40)
		≥$100,000		1 (6.7)
		Prefer not to answer		2 (13.3)
	**Relationship to person with dementia**			
		Spouse or partner		5 (33.3)
		Adult child		7 (46.7)
		Grandchild		2 (13.3)
		Other family member		1 (6.7)
	**Caregiving characteristics**			
		Sole caregiver		12 (80)
	**Duration of care**			
		6-12 months		2 (13.3)
		≥1 year		11 (73.3)
		Prefer not to answer		2 (13.3)
**Staff demographics (n=3)**				
	**Race (Ethnicity)**			
		Non-Hispanic White		2 (66.7)
		Non-Hispanic Black or African American		1 (33.3)
	**Sex**			
		Female		3 (100)

### Caregiver Confidence With Complex Care

Across 15 respondents, confidence was generally moderate to high for managing common dementia-related challenges (Table 4). For agitation, 10/15 (66.7%) reported being extremely or moderately confident; for mood changes, 10/15 (66.7%); for mental status changes, 12/15 (80%). Confidence in deciding whether to call a provider was high (13/15, 86.7% extremely or moderately). Communication-related competencies were strongest: 14/15 (93.3%) felt extremely or moderately confident talking to a medical provider, and 12/15 (80%) reported they could readily gather information a provider would want. Areas with more dispersion included understanding vital signs (10/15, 66.7% extremely or moderately; 3/15, 20% slightly) and assessing pain (9/15, 60% extremely or moderately).

**Table 4:** Caregiver confidence: frequency counts and median scores (n=15). Scores are based on a 5-point scale: 5=Extremely, 4=Moderately, 3=Somewhat, 2=Slightly, 1=Not at All. Median scores are reported alongside frequency counts.

Item			Extremely (5), n (%)		Moderately (4), n (%)		Somewhat (3), n (%)		Slightly (2), n (%)		Not at all (1),n (%)		Unknown			Median (IQR; 25th - 75th percentile)
**Managing symptoms and behaviors**																
	Dementia-related behaviors (ie, agitation)	2 (13.3)		8 (53.3)		3 (20)		1 (6.6)		1 (6.6)		0 (0)			4 (3-4)	
	Mood-related changes (ie, depression)	1 (6.6)		9 (60)		4 (26.6)		0 (0)		1 (6.6)		0 (0)			4 (3-4)	
	Mental status changes (ie, confusion)	6 (40)		6 (40)		2 (13.3)		1 (6.6)		0 (0)		0 (0)			4 (4-5)	
	New medical problems or events	3 (20)		8 (53.3)		2 (13.3)		0 (0)		2 (13.3)		0 (0)			4 (3.5-4)	
	Ongoing chronic diseases	3 (20)		7 (46)		3 (20)		1 (6.6)		1 (6.6)		0 (0)			4 (3-4)	
**Decision-making ability**																
	Dementia-related behaviors	2 (13.3)		8 (53.3)		3 (20)		0		2 (13.3)		0			4 (3-4)	
	Mood changes	1 (6.6)		7 (46.6)		4 (26.6)		2 (13.3)		1 (6.6)		0 (0)			4 (3-4)	
	Mental status changes	2 (13.3)		8 (53.3)		2 (13.3)		3 (20)		0 (0)		0 (0)			4 (3-4)	
	New medical problems	3 (20)		5 (33.3)		5 (33.3)		2 (13.3)		0 (0)		0 (0)			4 (3-4)	
	Ongoing chronic diseases	3 (20)		3 (20)		5 (33.3)		2 (13.3)		1 (6.6)		1 (6.6)			3 (3-4)	
**Health care communication**																
	Deciding whether or not to call the provider	4 (26.6)		9 (60)		2 (13.3)		0 (0)		0 (0)		0 (0)		4 (4-4.5)		
	Gathering information that a medical provider would want	8 (53.3)		4 (26.6)		3 (20)		0 (0)		0 (0)		0 (0)		5 (4-5)		
	Talking to a medical provider	10 (66.6)		4 (26.6)		1 (6.6)		0 (0)		0 (0)		0 (0)		5 (4-5)		
**Clinical assessment skills**																
	Taking vital signs	8 (53.3)		1 (6.6)		1 (6.6)		3 (20)		2 (13.3)		0 (0)		5 (2-5)		
	Understanding vital signs	6 (40)		4 (26.6)		1 (6.6)		1 (6.6)		3 (20)		0 (0)		4 (2.5-5)		
	Assessing for pain	3 (20)		6 (40)		3 (20)		3 (20)		0 (0)		0 (0)		4 (3-4)		
	Taking action when someone is in pain	5 (33.3)		5 (33.3)		3 (20)		2 (13.3)		0 (0)		0 (0)		4 (3-5)		
	Assessing for dehydration	3 (20)		5 (33.3)		3 (20)		2 (13.3)		1 (6.6)		1 (6.6)		4 (3-4)		
	Managing dehydration	5 (33.3)		4 (26.6)		4 (26.6)		2 (13.3)		0 (0)		0 (0)		4 (3-5)		
	Managing medical problems at home	4 (26.6)		5 (33.3)		3 (20)		2 (13.3)		1 (6.6)		0 (0)		4 (3-4.5)		

### People Living With Dementia Clinical Stage and Health Care Use

Care recipients were primarily in moderate to later stages of dementia: 7/15 (46.7%) had moderately severe cognitive decline, 2/15 (13.3%) severe, and 2/15 (13.3%) very severe. Within the prior 6 months, 6/15 (40%) had at least one emergency department visit, and 4/15 (26.7%) had been hospitalized. Medication burden was substantial: 10/15 (66.7%) were taking ≥5 daily medications and 4/15 (26.7%) were taking 3-5 (Table 5).

**Table 5:** People living with dementia: disease stage and health care (n=15).

Characteristics		Values, n (%)
**Stage of dementia**		
	Early-stage	4 (26.7)
	Moderate-severe cognitive decline	7 (46.7)
	Severe cognitive decline	2 (13.3)
	Very severe cognitive decline	2 (13.3)
**Health care use (past 12 months)**		
	Mean number of specialists seen	4 (26.7)
**Health care use (Past 6 months)**		
	Emergency department visit	6 (40)
	Hospitalization	4 (26.7)
**Total daily medications**		
	1-2 medications	1 (6.7)
	3-5 medications	4 (26.7)
	>5 medications	10 (66.7)

### Attrition and Engagement

Of the 15 caregivers who volunteered for the study, 14 logged into the app at least once, demonstrating high initial engagement. Four participants did not complete the study due to their loved one leaving the center, either due to death or requiring a higher level of care. Of the remaining caregivers, 8/10 completed the usability questionnaire and 6/10 participated in the follow-up interview. Among the 3 staff who participated, all (100%) logged into the app, completed the PSSQ, and completed the follow-up interview.

### App Usability and Perceived Impact on Access and Communication

Among the 8 caregivers who completed the application usability questionnaire, perceived usability and satisfaction were high. At least three-quarters “agreed/strongly agreed” that the app was easy to use (6/8, 75%) and easy to learn (7/8, 87.5%); 6/8 (75%) liked the interface and found information well organized. Most felt comfortable using the app in social settings (6/8, 75%) and endorsed appropriate time demands (6/8, 75%). Willingness to use the app beyond the study period was high (6/8, 75%), and overall satisfaction was favorable (6/8, 75% “agree/strongly agree”).

Communication and access benefits were frequently endorsed: 7/8 (87.5%) reported that the app improved access to their loved one at the center, 7/8 (87.5%) said it made communication with the ADC convenient, and 6/8 (75%) felt comfortable communicating with the center via the app. Navigation consistency was also rated positively (7/8, 87.5%) (Table 6).

**Table 6:** Application usability among caregivers (n=8)^a^.

Item, CareMobi app	Strongly Disagree (1), n (%)	Disagree (2), n (%)	Somewhat Disagree (3), n (%)	Neither Agree nor Disagree (4), n (%)	Somewhat Agree (5), n (%)	Agree (6), n (%)	Strongly Agree (7), n (%)	Median (IQR)
Easy to use	0 (0)	0 (0)	2 (25)	0 (0)	0 (0)	3 (37.5)	3 (37.5)	6 (5-7)
Easy to learn to use	0 (0)	0 (0)	1 (12.5)	0 (0)	2 (25)	2 (25)	3 (37.5)	6.5 (5.75-7)
Liked the interface	0 (0)	0 (0)	1 (12.5)	0 (0)	1 (12.5)	2 (25)	4 (50)	6.5 (5.75-7)
The information was well organized	0 (0)	0 (0)	1 (12.5)	0 (0)	1 (12.5)	2 (25)	4 (50)	6 (5.75-7)
Comfortable using in social settings	0 (0)	0 (0)	0 (0)	0 (0)	2 (25)	3 (37.5)	3 (37.5)	6 (5.75-6.25)
The amount of time involved in using it has been fitting for me	0 (0)	1 (12.5)	0 (0)	0 (0)	1 (12.5)	4 (50)	2 (25)	6 (5.75-6.25)
Would use it again	0 (0)	0 (0)	1 (12.5)	0 (0)	1 (12.5)	4 (50)	2 (25)	6 (5.75-7)
Overall, I am satisfied with it	0 (0)	0 (0)	0 (0)	1 (12.5)	1 (12.5)	3 (37.5)	3 (37.5)	4.5 (4-6)
Whenever I made a mistake using it, I could recover easily and quickly	0 (0)	0 (0)	1 (12.5)	3 (37.5)	1 (12.5)	2 (25)	1 (12.5)	6 (4.75-7)
It provides an acceptable way to send or receive health care communications	0 (0)	0 (0)	0 (0)	2 (25)	1 (12.5)	2 (25)	3 (37.5)	6 (5.75-7)
It adequately acknowledged and provided information to let me know the progress of my action	0 (0)	0 (0)	0 (0)	1 (12.5)	1 (12.5)	3 (37.5)	3 (37.5)	6 (6-7)
The navigation was consistent when moving between screens	0 (0)	0 (0)	0 (0)	1 (12.5)	0 (0)	4 (50)	3 (37.5)	6 (5.5-7)
The interface allowed me to use all the functions offered by the app (such as entering information, responding to notifications, viewing information)	0 (0)	0 (0)	0 (0)	2 (25)	0 (0)	3 (37.5)	3 (37.5)	6 (6-6)
It has all the functions and capabilities I expected it to have	0 (0)	0 (0)	0 (0)	1 (12.5)	0 (0)	6 (75)	1 (12.5)	6 (6-7)
It would be useful for my loved one’s well-being	0 (0)	0 (0)	0 (0)	0 (0)	0 (0)	5 (62.5)	3 (37.5)	6.5 (5.75-7)
It improved my access to my loved one in the adult day center	0 (0)	0 (0)	0 (0)	1 (12.5)	1 (12.5)	2 (25)	4 (50)	6 (4.75-7)
It helped me manage my loved one’s health effectively	0 (0)	0 (0)	0 (0)	2 (25)	1 (12.5)	2 (25)	3 (37.5)	6.5 (5.75-7)
It made it convenient for me to communicate with my adult day center	0 (0)	1 (12.5)	0 (0)	0 (0)	1 (12.5)	2 (25)	4 (50)	6 (5.75-6.25)
I had more opportunities to interact with adult day center staff	0 (0)	0 (0)	1 (12.5)	0 (0)	1 (12.5)	4 (50)	2 (25)	6 (5.75-7)
I felt confident that any information I send to a caregiver would be received.	0 (0)	1 (12.5)	0 (0)	0 (0)	1 (12.5)	3 (37.5)	3 (37.5)	7 (5.75-7)
I felt comfortable communicating with my adult day center using it	0 (0)	1 (12.5)	0 (0)	0 (0)	1 (12.5)	1 (12.5)	5 (62.5)	—^b^

^a^Scores are based on a 7-point scale: 7=Strongly Agree, 6=Agree, 5=Somewhat Agree, 4=Neither Agree nor Disagree, 3=Somewhat Disagree, 2=Disagree, 1=Strongly Disagree. Median scores are reported alongside frequency counts.

^b^Not applicable.

### Staff Usability of the CareMobi App

Staff reported highly favorable usability ratings, with median scores at or near the ceiling across nearly all items (median=7, “strongly agree”). All 3 participants indicated that the CareMobi app was easy to use, easy to learn, and well organized (median=7). Satisfaction was uniformly high (all median=7): staff strongly agreed they would use the app again, that it contained the functions and capabilities expected, and that it provided an acceptable way to communicate with caregivers.

Communication-related items also demonstrated the strongest endorsement. Staff unanimously reported that the app improved access to caregivers, made communication more convenient, and increased opportunities to interact with caregivers (median=7 across items). They also expressed confidence that any information sent would be reliably received (median=7).

Minor variability was observed on a small number of items. For example, one staff member reported difficulty recovering from mistakes, producing a median score of 6 (“agree”) on that item. Another staff member selected “neither agree nor disagree” on certain workflow-related items (eg, time fit and error recovery), though the median across sites still remained 7 (Table 7).

**Table 7:** Staff application usability—combined results (n=3).

Item, CareMobi app	Strongly Disagree (1), n (%)	Disagree (2), n (%)	Somewhat Disagree (3), n (%)	Neither Agree nor Disagree (4), n (%)	Somewhat Agree (5), n (%)	Agree (6), n (%)	Strongly Agree (7), n (%)	Median (IQR)
Easy to use	0 (0)	0 (0)	0 (0)	0 (0)	0 (0)	0 (0)	3 (100)	7 (7-7)
Easy to learn to use	0 (0)	0 (0)	0 (0)	0 (0)	0 (0)	0 (0)	3 (100)	7 (7-7)
Liked the interface	0 (0)	0 (0)	0 (0)	0 (0)	0 (0)	0 (0)	3 (100)	7 (7-7)
The information was well organized	0 (0)	0 (0)	0 (0)	0 (0)	0 (0)	0 (0)	3 (100)	7 (7-7)
Comfortable using in social settings	0 (0)	0 (0)	0 (0)	0 (0)	0 (0)	0 (0)	3 (100)	7 (5.5-7)
The amount of time involved in using it has been fitting for me	0 (0)	0 (0)	0 (0)	1 (33.3)	0 (0)	0 (0)	2 (66.6)	7 (7-7)
Would use it again	0 (0)	0 (0)	0 (0)	0 (0)	0 (0)	0 (0)	3 (100)	7 (7-7)
Overall, I am satisfied with it	0 (0)	0 (0)	0 (0)	0 (0)	0 (0)	0 (0)	3 (100)	4 (3-5.5)
Whenever I made a mistake using it, I could recover easily and quickly	0 (0)	1 (33.3)	0 (0)	1 (33.3)	0 (0)	0 (0)	1 (33.3)	7 (7-7)
It provides an acceptable way to send or receive health care communications	0 (0)	0 (0)	0 (0)	0 (0)	0 (0)	0 (0)	3 (100)	7 (7-7)
It adequately acknowledged and provided information to let me know the progress of my action	0 (0)	0 (0)	0 (0)	0 (0)	0 (0)	0 (0)	3 (100)	7 (6.5-7)
The navigation was consistent when moving between screens	0 (0)	0 (0)	0 (0)	0 (0)	0 (0)	1 (33.3)	2 (66.6)	7 (5.5-7)
The interface allowed me to use all the functions offered by the app (such as entering information, responding to notifications, viewing information)	0 (0)	0 (0)	0 (0)	1 (33.3)	0 (0)	0 (0)	2 (66.6)	6 (5.5-6.5)
It has all the functions and capabilities I expected it to have	0 (0)	0 (0)	0 (0)	0 (0)	1 (33.3)	1 (33.3)	1 (33.3)	7 (7-7)
It would be useful for my loved one’s well being	0 (0)	0 (0)	0 (0)	0 (0)	0 (0)	0 (0)	3 (100)	7 (5.5-7)
It improved my access to my loved one in the adult day center	0 (0)	0 (0)	0 (0)	1 (33.3)	0 (0)	0 (0)	2 (66.6)	7 (7-7)
It helped me manage my loved one’s health effectively	0 (0)	0 (0)	0 (0)	0 (0)	0 (0)	0 (0)	3 (100)	7 (7-7)
It made it convenient for me to communicate with my adult day center	0 (0)	0 (0)	0 (0)	0 (0)	0 (0)	0 (0)	3 (100)	7 (5.5-7)
I had more opportunities to interact with adult day center staff	0 (0)	0 (0)	0 (0)	1 (33.3)	0 (0)	0 (0)	2 (66.6)	7 (6-7)
I felt confident that any information I send to a caregiver would be received.	0 (0)	0 (0)	0 (0)	0 (0)	1 (33.3)	0 (0)	2 (66.6)	7 (6.5-7)
I felt comfortable communicating with my adult day center using it	0 (0)	0 (0)	0 (0)	0 (0)	0 (0)	1 (33.3)	2 (66.6)	—^a^

^a^Not applicable.

### Explanatory Qualitative Findings (Caregivers and Staff)

Consistent with the high ratings for convenient communication and improved access (75-88%), interviews with caregivers and staff, and open-ended written comments, revealed eight major themes that described CareMobi’s value and limitations in daily practice.

In general, caregivers expressed strong support for CareMobi in their open-ended survey responses: “CareMobi is very helpful to me.” “It is great.” “Keep it up :)” “I found the app to be user friendly and easy to communicate to others.”

#### Transparency

Caregivers valued the app’s ability to provide “visibility into the day” reducing uncertainty about what occurred at the center and allowing them to interpret behaviors at home. One staff member shared: “If they’re acting different, then we may know it’s because they didn’t get a good night rest.” Family caregivers described using updates to understand changes in appetite and weight: “He eats all the stuff at the center he won’t touch at home. Seeing that helped me realize the weight loss wasn’t from not eating at all.” Another wrote, “This app gives caregivers the comfort of knowing they are a tap away from knowing the daily report of their loved ones.”

#### Streamlined Communication and Time Savings

Staff and caregivers highlighted that CareMobi replaced “phone tag” with more efficient messaging. A staff member explained: “Better communication between our center and the families… We can just put it in CareMobi and communicate that way.” Staff also emphasized the time saved: “Instead of spending half the morning calling families one by one, I can send the update once in the app.” This freed staff to focus more on direct care tasks.

#### Guidance on What to Track

Both groups requested clearer guidance on what domains to log. Caregivers wanted consistent updates on hydration and bathroom use, saying “It’s interesting that we don’t track that in here.” Staff asked for templates (eg, “Today at a Glance,” “Transport Update”) to reduce duplication and ensure the right staff member provided the right update. As one caregiver reflected:

#### Value Across the Care Trajectory

Staff reported the app was particularly helpful for new enrollees, giving families reassurance and setting expectations from the start. One staff member noted: “Really helpful with the new enrollees, because they don’t know any different.” While families of newly enrolled clients used it to communicate with center staff, established caregivers used it more to maintain daily stability and track ongoing concerns.

#### Technology Fit and Integration

While usability was generally positive, staff emphasized the need to minimize duplicate data entry and expressed interest in a web or desktop option. One explained: “[Caregivers] have their own portals and our own things you’re using.” This theme mirrored broader structural challenges, as many ADCs lack interoperable EHRs and rely on paper or billing software.

#### Operational Constraints

Participation was shaped by staffing capacity and caregiver demands. Rural centers with small teams were able to sustain use; however, caregivers cited competing responsibilities: “I’m juggling work and his appointments, some days I just can’t get to it.” One caregiver noted they still relied on phone calls for reassurance: “I have great communication with [the center] by phone. If any problems occur I know they will contact me. I also know I can contact them by phone.”

#### Trust, Confidence, and Relationship-Building

Routine updates fostered trust and provided caregivers with peace of mind. One caregiver explained: “The better you know how things are going throughout the day, the better the day is for you both.” Importantly, transparency also strengthened caregivers’ confidence in communicating with physicians: “When I went to the doctor, I could show them what was happening at the center. I felt more prepared.” Multiple caregivers described using CareMobi data to adjust medications: “We actually got him off one of his meds, because I could prove he was eating and drinking fine there.” Staff echoed that regular updates improved caregiver satisfaction and contributed to steadier enrollment: “This app will give caregivers the comfort of knowing they are a tap away from knowing the daily report of their loved ones.”

## Discussion

### Principal Findings

This study evaluated the acceptability, demand, and practicality of CareMobi, a mobile platform designed to strengthen communication between ADCs and family caregivers. We posit that by making ADC-based care more transparent to families (through timely updates and role-specific, low-burden interactions), CareMobi, if feasible and acceptable, can reduce uncertainty and build trust among caregivers. This, in turn, can support sustained participation in ADCs and bolster engagement and connectedness for people living with dementia who are at risk of loneliness and social isolation. Our findings provide preliminary support for this proposition.

### Acceptability and Satisfaction

CareMobi demonstrated high acceptability among both caregivers and staff, meeting or exceeding all prespecified STOP-AMEND-GO thresholds [17]. Specifically, usability scores surpassed the >75% “agree/strongly agree” criterion across all items, satisfaction levels exceeded benchmarks, and more than 70% of caregivers engaged with the app at least once. Staff usability scores reached ceiling levels, with median ratings of 7 (strongly agree) across nearly all items. Acceptability was also met, with more than three-quarters of participants expressing interest in using CareMobi beyond the study. Caregivers emphasized the platform was “user friendly,” “very helpful,” and reassured them by being “a tap away from knowing the daily report of their loved ones.” Together, these findings provide strong evidence of acceptability and support CareMobi’s potential as a low-burden, scalable tool for use in ADCs, and provide robust evidence for moving forward with broader implementation trials.

### Demand and Engagement

Technology adoption in dementia caregiving populations has historically been low, with many studies reporting attrition, underuse, or abandonment of digital tools due to steep learning curves, lack of personalization, or misalignment with caregivers’ daily realities [18,19]. In contrast, CareMobi achieved high engagement among study volunteers: 93% of caregivers logged into the app at least once, and 75% of caregivers and 100% of staff expressed willingness to continue use beyond the study. This success may be attributed to its co-designed, user-centered development process, which emphasized simplicity. Staff specifically highlighted reduced “phone tag,” noting that streamlined messaging saved time and allowed them to redirect effort toward direct care. This represents an important advantage over other health care technologies that inadvertently add to staff burden rather than reducing it [12]. **Staff observed that bidirectional communication via the app was more likely to be achieved with caregivers who were new to the ADC, as they relied on CareMobi as their primary means of communication with staff. In contrast, established caregivers, who were more accustomed to phone-based communication, tended to use the app more passively to track updates. This suggests that** caregivers who have developed routines with staff may perceive less added value in switching.

It is worth noting that our sample was composed of caregivers who reported relatively high baseline confidence in managing their loved ones’ needs, which may have facilitated their willingness to engage with a new tool. Many were also sole caregivers, relying on the ADC as an extension of their kinship network. This close relational tie to the center may have further motivated consistent use of CareMobi, as research suggests that engagement is supported not only by the design of the technology but also by the social context in which it was deployed [20].

### Implementation and Practicality

ADC staff were able to integrate CareMobi into their workflows, but several refinements were identified. Both caregivers and staff requested more structured guidance on which observations should be logged (eg, hydration, bathroom trips, and mood changes) and templates such as “Today at a Glance” or “Medication Updates” to enhance efficiency. Staff expressed demand for a desktop or web version since most entered data from a computer, as opposed to a smartphone or tablet. Research shows that community-based settings, especially those with limited resources, are often excluded from technological innovation and struggle because of a fragmented digital infrastructure [21,22]. By focusing on low-cost, high-utility design, and implementing requested refinements, CareMobi can help address these structural inequities, ensuring that centers with fewer resources are not left behind in digital health advancement.

### Caregiver Confidence and Trust

Another critical finding was CareMobi’s impact on caregiver confidence and trust. Families reported using real-time updates to interpret behaviors at home, guide conversations with medical providers, and even advocate for deprescribing when daily logs showed adequate eating and drinking. One caregiver shared: “We actually got him off one of his meds, because I could prove he was eating and drinking fine there.” This echoes existing evidence that improved caregiver communication can enhance self-efficacy, reduce decisional conflict, and promote timely care coordination [23,24]. By strengthening transparency, CareMobi appears to foster not only trust in ADCs but also caregiver empowerment in broader health care decision-making. As another caregiver said, “The better you know how things are going throughout the day, the better the day is for you both.”

### Equity and Representation

Although findings were promising, the sample was predominantly Non-Hispanic White, female, and English-speaking. This does not reflect the broader demographics of ADC families nationally, where approximately 58% of clients identify as racial or ethnic minorities [4]. Language accessibility was a major limitation; CareMobi is not available in Spanish or other languages. This restricted participation for centers serving diverse populations. This represents both a limitation of the current evaluation and a critical direction for future work. Addressing language and cultural tailoring is essential for scaling CareMobi equitably and ensuring its benefits extend to the diverse populations that rely most on adult day services.

### Broader Implications

Collectively, these findings suggest that CareMobi can address longstanding barriers to ADC use. By improving “day-visibility,” simplifying communication, and providing structured yet flexible updates, the app has the potential to stabilize attendance and support sustained participation, barriers that have historically undermined ADCs’ ability to demonstrate value in long-term care [25,26]. This is particularly timely given the underfunding of adult day services and their critical role in providing cost-effective, community-based care for people with dementia [7].

### Limitations and Future Directions

While this pilot yielded encouraging results, this study had several limitations. First, although appropriate for a feasibility design, the sample size was small and drawn from 2 centers, limiting generalizability. The sample was also demographically homogenous (ie, primarily Non-Hispanic White, female, and English-speaking) and did not reflect the diversity of families served by ADCs nationally. As participation required English proficiency and smartphone ownership, findings may overrepresent caregivers who were more comfortable with technology and positively predisposed to digital tools. Second, the study duration was relatively short, constraining our ability to draw conclusions about long-term engagement or patient outcomes. Third, while we evaluated usability, satisfaction, and acceptability, we did not measure downstream effects like caregiver burden or health care use. These outcomes will be the focus of future trials.

The findings from this feasibility study will directly inform the next phase of research, which will involve a larger, multisite hybrid effectiveness–implementation trial. Future work will assess whether improved communication and transparency translate into measurable outcomes such as reductions in caregiver burden, improved people living with dementia engagement and quality of life, more consistent ADC attendance, and reduced acute-care use. Recruitment will intentionally prioritize racially, ethnically, and linguistically diverse caregivers and centers, including development of multilingual platform versions and community-engaged recruitment strategies.”

### Conclusions

By coupling digital transparency with low-burden communication, CareMobi offers a promising approach to modernizing ADCs in ways that respect resource constraints and caregiver realities. In doing so, it reduces caregivers’ uncertainty about sending people living with dementia to the ADC, in turn, providing the people living with dementia an opportunity to socialize and engage. To fully realize this potential, future work must prioritize equity, especially linguistic inclusion, infrastructure support, and engagement in under-resourced settings. Ultimately, if scaled thoughtfully, tools like CareMobi may enrich the social fabric of dementia care, increase accessibility of ADCs, and deepen the connection between caregivers, centers, and people living with dementia.

## Data Availability

The data are unavailable to access publicly due to privacy and ethical restrictions.

## Abbreviations

ADC: adult day center
CCSM: Caregiver Confidence in Sign/Symptom Management Scale
HIPAA: Health Insurance Portability and Accountability Act
mHealth: mobile health
PSSUQ: Post-Study System Usability Questionnaire
